# Supplementary figures and images for: COVID-19 and acute kidney injury in German hospitals 2020
Source: PLoS One. 2022 Jun 9;17(6):e0264510. doi: 10.1371/journal.pone.0264510 (PMC9182294; doi:10.1371/journal.pone.0264510)

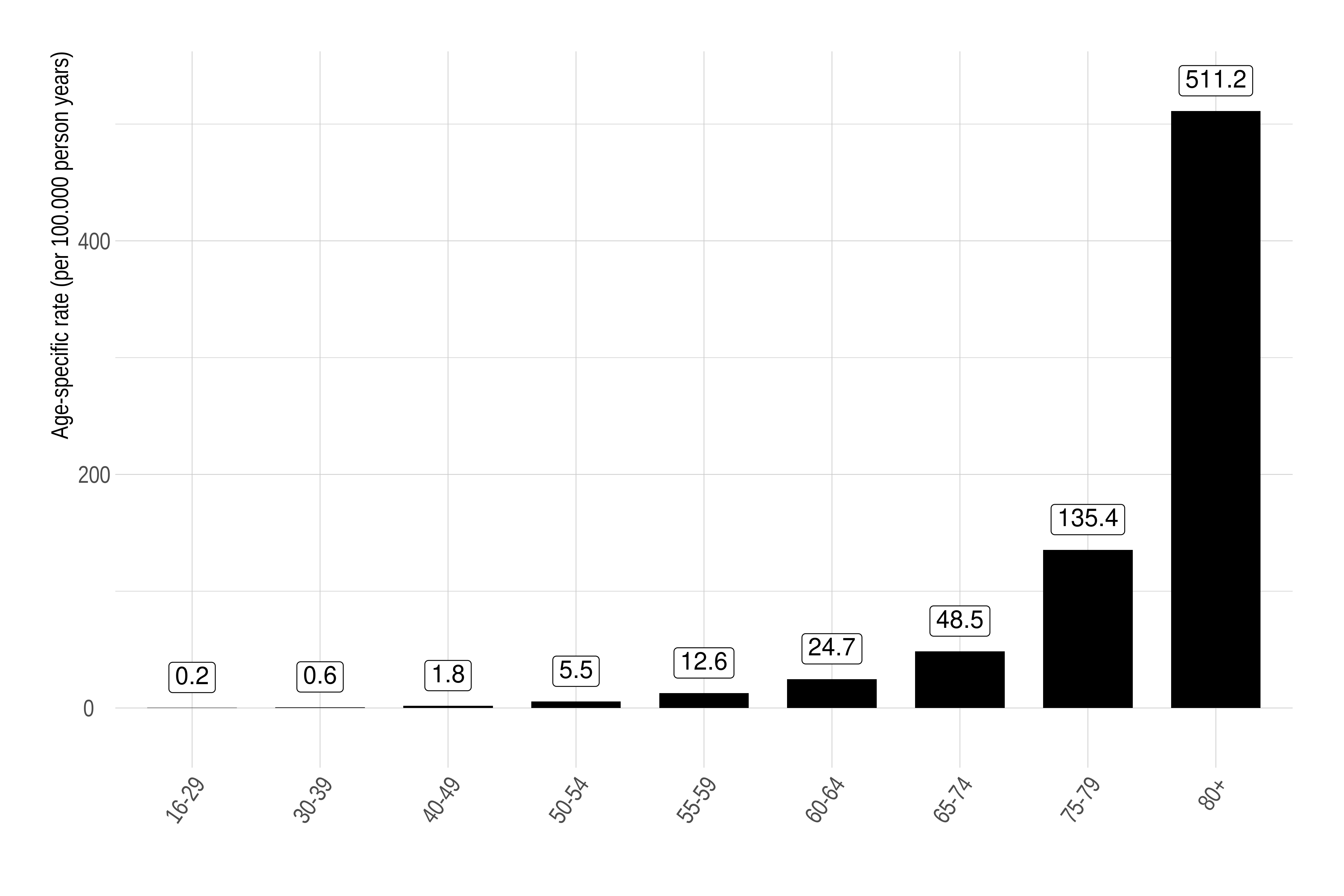

Supplement: S1 Fig — (TIFF) [file pone.0264510.s001.tiff]

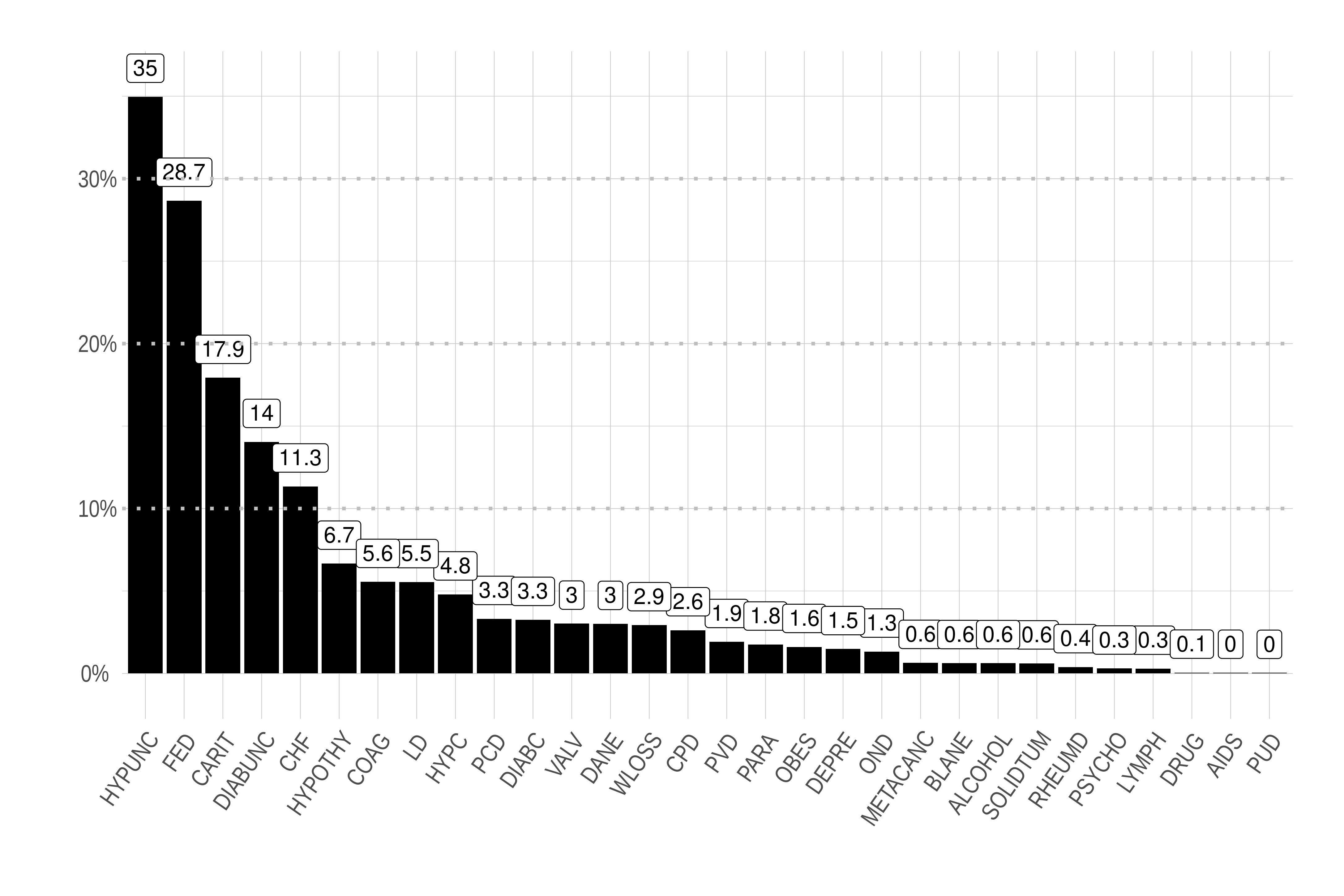

Supplement: S2 Fig — chf: congestive heart failure; carit: cardiac arrhythmias; valv: valvular disease; pcd: pulmonary circulation disorders; pvd: peripheral vascular disorders; hypunc: hypertension—uncomplicated; hypc: hypertension—complicated; para: paralysis; ond: other neurological disorders; cpd: chronic pulmonary disease; diabunc: diabetes -uncomplicated; diabc: diabetes- complicated; hypothy: hypothyroidism; ld: liver disease; pud: peptic ulcer disease- excluding bleeding; aids: AIDS/HIV; lymph: lymphoma; metacanc: metastatic cancer; solidtum: solid tumour: without metastasis; rheumd: rheumatoid arthritis/collaged vascular disease; coag: coagulopathy; obes: obesity; wloss: weight loss; fed: fluid and electrolyte disorders; blane: blood loss anaemia; dane: deficiency anaemia; alcohol: alcohol abuse; drug: drug abuse; psycho: psychoses; depre: depression. (TIFF) [file pone.0264510.s002.tiff]

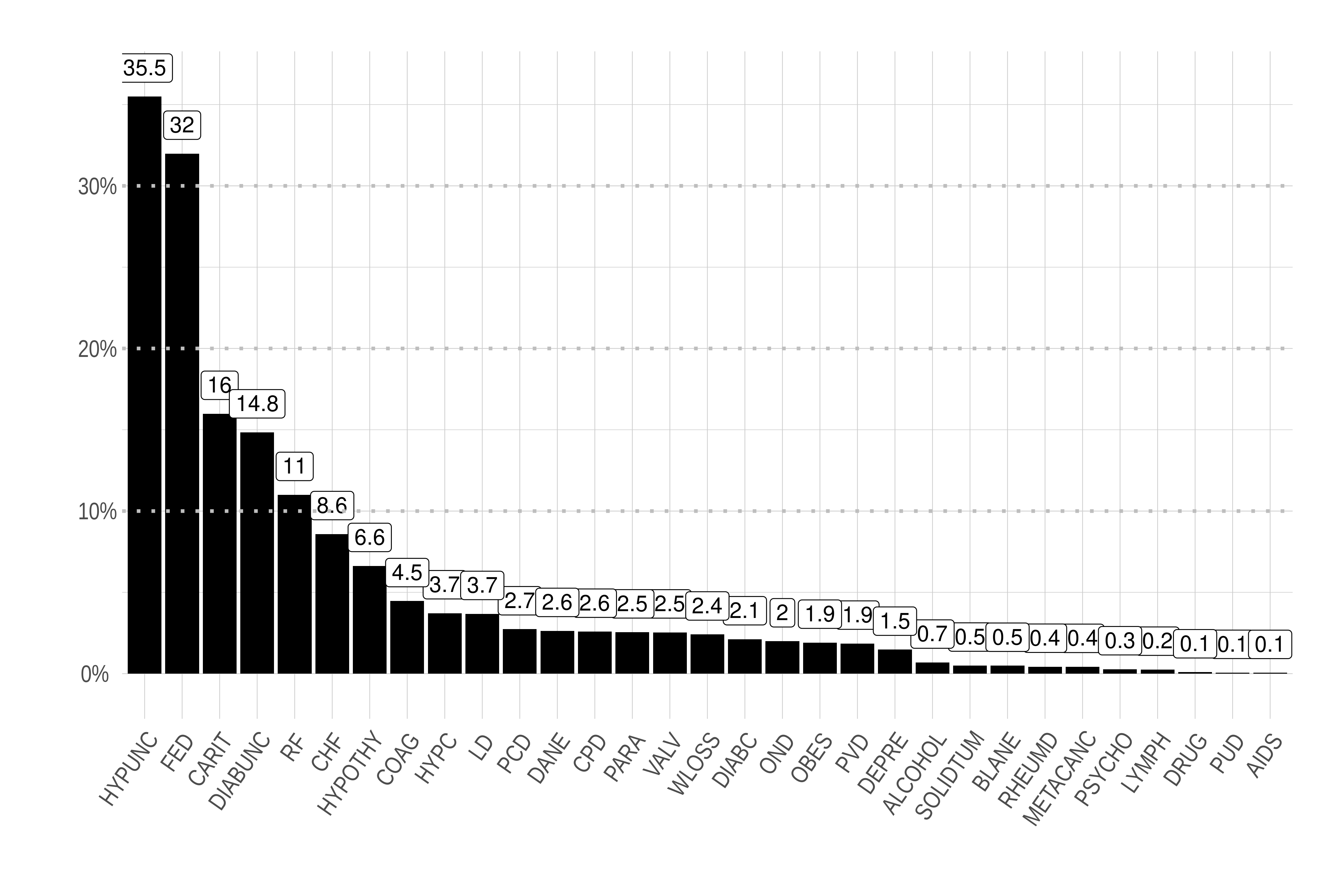

Supplement: S3 Fig — chf: congestive heart failure; carit: cardiac arrhythmias; valv: valvular disease; pcd: pulmonary circulation disorders; pvd: peripheral vascular disorders; hypunc: hypertension—uncomplicated; hypc: hypertension—complicated; para: paralysis; ond: other neurological disorders; cpd: chronic pulmonary disease; diabunc: diabetes -uncomplicated; diabc: diabetes- complicated; hypothy: hypothyroidism; ld: liver disease; pud: peptic ulcer disease- excluding bleeding; aids: AIDS/HIV; lymph: lymphoma; metacanc: metastatic cancer; solidtum: solid tumour: without metastasis; rheumd: rheumatoid arthritis/collaged vascular disease; coag: coagulopathy; obes: obesity; wloss: weight loss; fed: fluid and electrolyte disorders; blane: blood loss anaemia; dane: deficiency anaemia; alcohol: alcohol abuse; drug: drug abuse; psycho: psychoses; depre: depression. (TIFF) [file pone.0264510.s003.tiff]

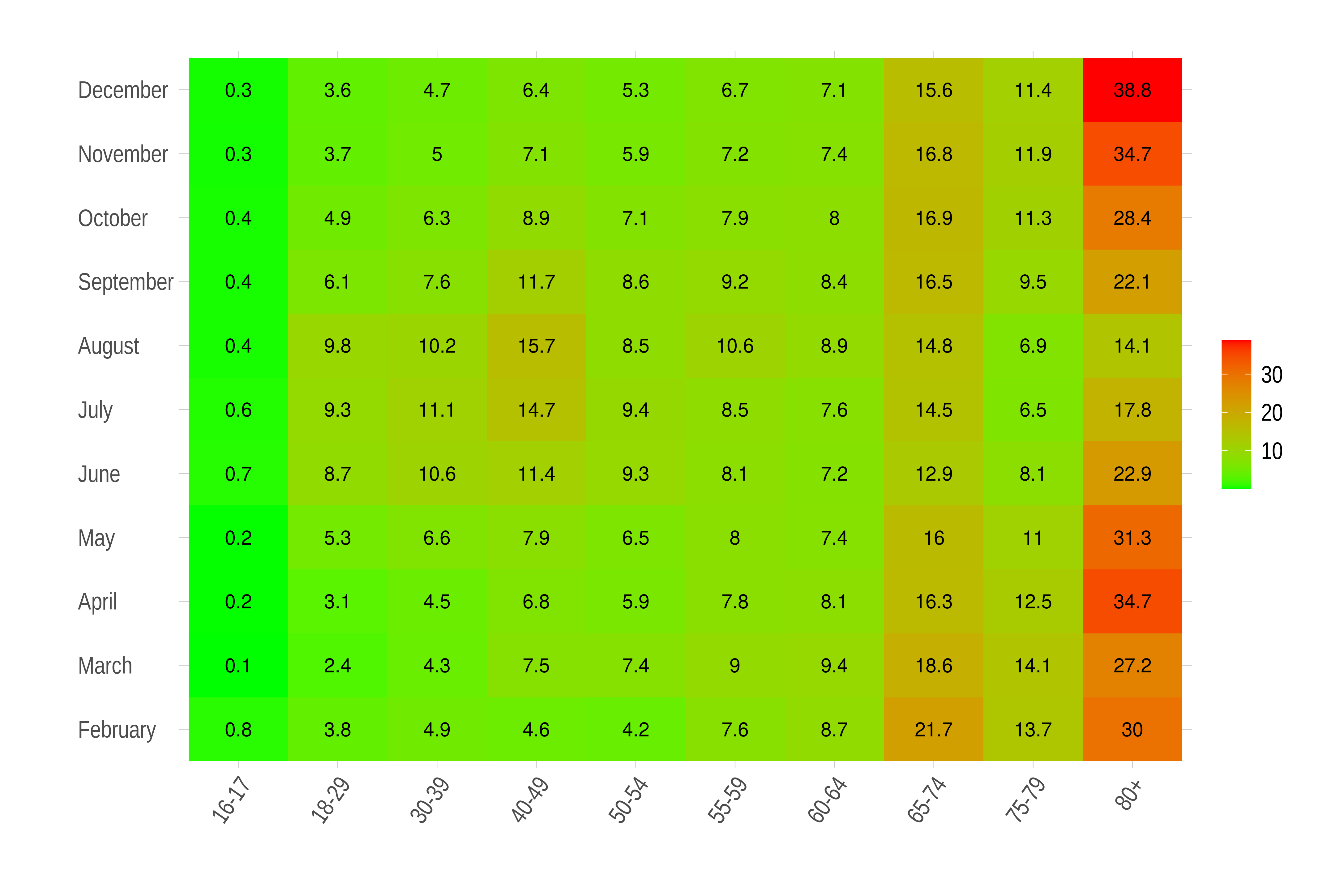

Supplement: S4 Fig — (TIFF) [file pone.0264510.s004.tiff]

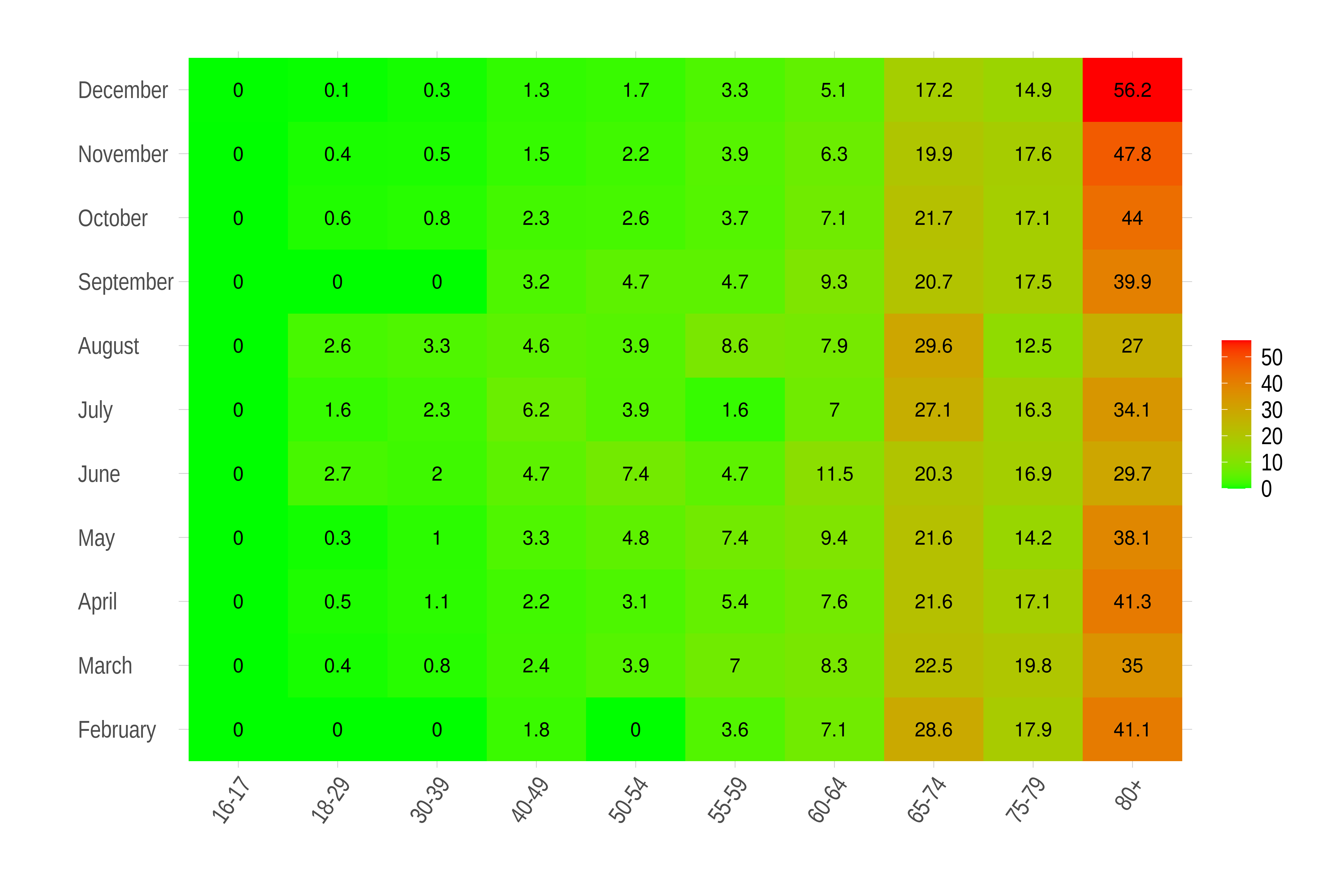

Supplement: S5 Fig — (TIFF) [file pone.0264510.s005.tiff]
